# State of vulnerable populations in the techquity framework in Hungary

**DOI:** 10.3389/fpubh.2023.1215325

**Published:** 2023-07-06

**Authors:** Zsuzsa Győrffy, Bence Döbrössy, Nóra Radó, Julianna Boros, Sándor Békási

**Affiliations:** ^1^Faculty of Medicine, Institute of Behavioural Sciences, Semmelweis University, Budapest, Hungary; ^2^DocRoom Health Research Program, Health Center, Hungarian Charity Service of the Order of Malta, Budapest, Hungary; ^3^Telemedicine Workgroup, FitPuli Kft, Győr, Hungary

**Keywords:** techquity, permacrisis, digital health, telemedicine, vulnerable populations, health equity

## Abstract

Digital health solutions could alleviate the needs of vulnerable populations in the recent period of the permacrisis, however, there are several barriers that limit their use for certain individuals. We use the four-pillar model of the novel concept of techquity to provide original evidence of the discrepancy in the willingness to try and the ability to harness healthtech in Hungary. We identified three underserved segments of society: older adults, people with long-term activity-limiting conditions, and people experiencing homelessness who could greatly benefit from digital technologies and yet use them less than the general population. We also discuss potential strategic considerations in order to promote techquity and digital inclusion among people living in vulnerable situations.

## 1. Introduction: techquity in the permacrisis

Perhaps the biggest milestones of digitization in recent decades have been the emergence of the Internet and the spread of smartphones. In the late ‘90s, the Internet took off but its unlimited application in everyday situations was driven by smartphones. A similar trend can be seen in digital health: before the COVID-19 pandemic, many digital health solutions were already available, from robotic surgery to sensors, but the pandemic gave a boost to the adoption of digital technologies.

However, technological potential is only one component of systematic change. For digital health solutions to be genuinely widespread, they require adaptation on both the patient and provider sides. Digital health is a cultural and social transformation of healthcare (1). It is important to make sure that the technology is accessible and usable to segments of the population where it is most needed but equally vital to shaping norms, values, knowledge, and beliefs.

As stated by the World Health Organization (WHO) Global strategy on digital health 2020–2025, *“Digital health should be an integral part of health priorities and benefit people in a way that is ethical, safe, secure, reliable, equitable and sustainable*” (2). Research tells us that those who we assume would benefit most from digital health, use them less (3). While the use of digital health solutions is increasing overall, well-defined segments of society are left out. As Tudor Hart observed over 50 years ago, the availability of good medical care tends to vary inversely with the need for it in the population served (4). This holds for digital health, too.

To describe the efforts to lessen societal inequalities via digital health tools, we use techquity as a new concept. Techquity is the *“intentional design and deployment of technology both to advance health equity and to avoid depending existing systemic inequities and health disparities”* (5–7). Techquity is built on the following pillars: trust in technology, access to, initial use/adoption of the technology, and sustained engagement with it. While the widely used term digital divide emphasizes the differences and loss of opportunities, the techquity framework represents a well-structured approach with more actionable insights by providing a step-by-step evaluation of digital tools. It underlines the role and responsibility of technology in producing social gradients in health and encourages deeper commitment to eliminating structural inequities (8).

In addition to techquity, the term permacrisis is also just gaining currency. It is defined by Collins Dictionary as an extended period of instability and insecurity (9). It reflects the fact that we are facing challenges like escalating climate change, the COVID-19 pandemic, the war in Ukraine, the energy crisis, and inflation all influencing the way we are able to utilize healthcare. Global production and supply chains have been in the process of breaking up for a while now (10). There are long-running health and social inequalities. Moreover, the pandemic was experienced much more severe among disadvantaged social groups both in terms of health and economic consequences (11). Digital health grew roots early in better-developed, wealthier countries but was underutilized in areas poorer in resources (12). During the pandemic, there was a sharp rise in its use in many countries (13).

In Hungary, the digital health revolution took place in just a few months of rapid policymaking related to the pandemic but the cultural transition is lagging behind (14). According to the Digital Economy and Society Development Index 2022 (DESI), Hungary is ranked 22nd among the 27 European Union member states. 49% of people have at least basic digital skills, significantly below the EU average of 54%, however, the country’s performance in broadband subscriptions, 5G spectrum and very high-capacity fixed network (VHCN) coverage exceeded the EU average (15).

In this perspective, we will provide original research evidence that digital health has the potential to reach and help underserved populations but without reinforcing efforts, the most in need will benefit the least. Without special attention given to issues of health equity, innovation in itself will further widen the accessibility gap instead of closing them.

## 2. Methodological framework

Our research team *Digital Health Solutions in Medicine* (Semmelweis University, Budapest, Hungary) recently conducted surveys that either included vulnerable or underserved individuals or focused exclusively on them. Within our previous study samples, we have identified three vulnerable subpopulations: older adults, people living with long-term activity-limiting impairment, and people experiencing homelessness for further evaluation. The detailed methodological description of each study is referred to as already available publications in this section. Relevant demography parameters of each group are added as Supplementary material.

In this paper, we provide a secondary analysis of data representing a unique insight into different well-identified groups, all affected by the struggles of digital inclusion. As the level of digital development, the economic status, and the utilization of healthcare services might significantly vary from country to country, the fact that all studies were completed on the Hungarian population gave the opportunity for a direct comparison of results coming from the same national background.

All our studies were approved by the Research Ethics Committee of the Hungarian Medical Research Council. The ethical license number is IV-10927-1/EKU.

### 2.1. Research data on older adults and people living with long-term activity-limiting conditions

A national telephone questionnaire survey among the Hungarian population focusing on digital health-related knowledge, attitudes, and needs was completed in 2021 (16). The sample size was 1,500 and it was representative of the adult population of Hungary in terms of gender, age, type of settlement, and education. From this sample, we identified two subpopulations for further analysis: respondents 65 years and older (*N* = 428) and people living with long-term activity-limiting conditions (*N* = 272) (17). For the latter, a single question of the Global Activity Limitation Indicator (GALI) was used (18). The overlap between these two groups was 38.8%. During the statistical data processing, distributions, cross-tabulations and chi-square tests were performed.

### 2.2. Research data on people experiencing homelessness

In collaboration with the DocRoom Health Research Program at the Hungarian Charity Service of the Order of Malta (Budapest, Hungary), we have been working together on numerous studies on the issue of how vulnerable groups such as people experiencing homelessness could benefit from digital health technologies. The three main aspects of the joint previous research projects were: attitudes and openness of people experiencing homelessness toward telemedicine (19), access to digital devices and health-related Internet use among people experiencing homelessness (20), and the evaluation of a telemedicine pilot project in homeless shelters (21). The number of participants in each study was N = 98, 662, and 75, respectively. In these studies, no overlaps with the previous two groups of the representative survey were identified.

### 2.3. Application of the techquity framework

The secondary analysis of our research data is presented in a form that represents the four-pillar model of techquity published by the HLTH Foundation (New York, NY, United States) in partnership with Ipsos (New York, NY, United States) in 2023 (7). In this current paper, we consider the manifestation of trust as relevant openness toward or willingness to try new technology (Pillar 1). For Pillar 2, we consider access to technology as access to digital resources such as the Internet, smartphones, tablets, computers, and other digital tools. Initial use or adoption of the technology (Pillar 3) was represented in our data as frequency measurements: those respondents who used a digital resource at least once a month were considered adopters of the technology. Due to methodological constraints, it was difficult to indicate a separate measurement for access to health technology, initial usage, and adoption of the technology, therefore we present combined data of Pillars 2 and 3 below regarding the three subpopulations. Under Pillar 4, we summarized data on self-defined recurrent or regular use of healthtech and compliance with telemedicine services.

## 3. Research findings on the pillars of techquity

### 3.1. Pillar 1: trust in the technology

Older adults reported significant interest in digital technologies: only a quarter of 65–74-year-olds (26.5%) and a third of 75 + −year-olds (31.9%) responded they would not like to try digital technologies in the future, while nearly 70% of both age groups would like to learn about such tools. The high level of openness was also shown by the fact that more than a fifth of older adults would like to have access to the maximum number of digital technologies mentioned in the survey (answer options were: online appointment bookings, e-prescriptions, data/information exchange with a physician, social media use for health purposes, mobile health applications, telemedicine, and smart devices for health monitoring).

In the case of people living with long-term activity-limiting conditions, when we asked about digital technologies they would like to use from those they had not tried yet, they were less open regarding all of the possibilities compared to the answers of people with no limitations. People living with long-term activity-limiting conditions seemed to be more pessimistic, they reported finding fewer benefits and more limitations to digital technologies. There was a significant difference in the number of benefits of digital solutions mentioned (people living with long-term activity-limiting conditions: mean = 7.4, n = 272, reference group: mean = 7.7, *n* = 1,228; *p* = 0.02) and the number of perceived disadvantages as well (people living with long-term activity-limiting conditions: mean = 6.1, n = 272, reference group: mean = 5.6, *n* = 1,228; *p* = 0.005).

Comparing the attitude toward telemedicine, the responses of people experiencing homelessness and a national reference group revealed that the index population was not differing from the general public, their openness was equal on a 5-point Likert scale. However, homeless individuals who felt that they received adequate care and attention were more likely to believe that telemedicine was suitable for them as a form of care. Openness regarding online visits can primarily be based on trust in the healthcare system. The WHO considers trust to be extremely important in the implementation of digital health (2), and this was also confirmed by our research. While in other countries trust in technology, data protection, and security is the main focus of the general population, for people experiencing homelessness, trust seemed to be more related to the global trustworthiness of the healthcare system in Hungary.

### 3.2. Pillars 2 and 3: access to, initial use or adoption of the technology

43.9% of older adults accessed the Internet at least once a month (as compared to 81.3% of the total population) and they were significantly less likely to search for health information online as well. This remarkable difference was seen in the use of other digital health solutions, too. While 42.8% of the population booked medical appointments online, only 27.9% of older adults done so. Their use of e-prescriptions was roughly 10 percentage points lower than that of the general population (76.4% vs. 86.4%). Older adults also used mobile health applications (14.3% vs. 27.3%) and smart devices or sensors (13.7% vs. 37.3%) less.

People living with long-term activity-limiting conditions reported that digital technologies (such as emailing, sharing of electronic health records, and online appointment booking) were less common in interactions with physicians. Digital transmission of health-related data was used by 58.5% of people with no activity limitations, 34.8% of people with mild activity limitations, and only 25.7% of people with severe limitations. Smart sensors were used by 32.8% of people with no activity limitations, 21.7% with mild limitations, and 17.6% of people with severe limitations. When we asked about the patients’ needs in connection with digital communication and device usage, people living with disabilities reported disadvantages in all aspects, both in technologies already used and in options that the respondents had not tried before but would like to use if they had access.

In the case of people experiencing homelessness, 52.9% of respondents said they used the Internet frequently, compared with 81.3% in the general population from the representative survey. Among the homeless group, 39.9% had a smartphone and 34.6% of the respondents had used the Internet for medical purposes, which was significantly less than in the general population (71.3%). However, we could identify a digitally engaged group, which we defined as people who used the Internet every two week or more frequently, accessed the Internet with their own device, rated themselves average or more competent Internet users, and used the Internet for health-related reasons. This group represented 19.5% of the surveyed population. Additionally, 11.2% of people experiencing homelessness had already used a mobile health application at least once.

**Figure 1:**
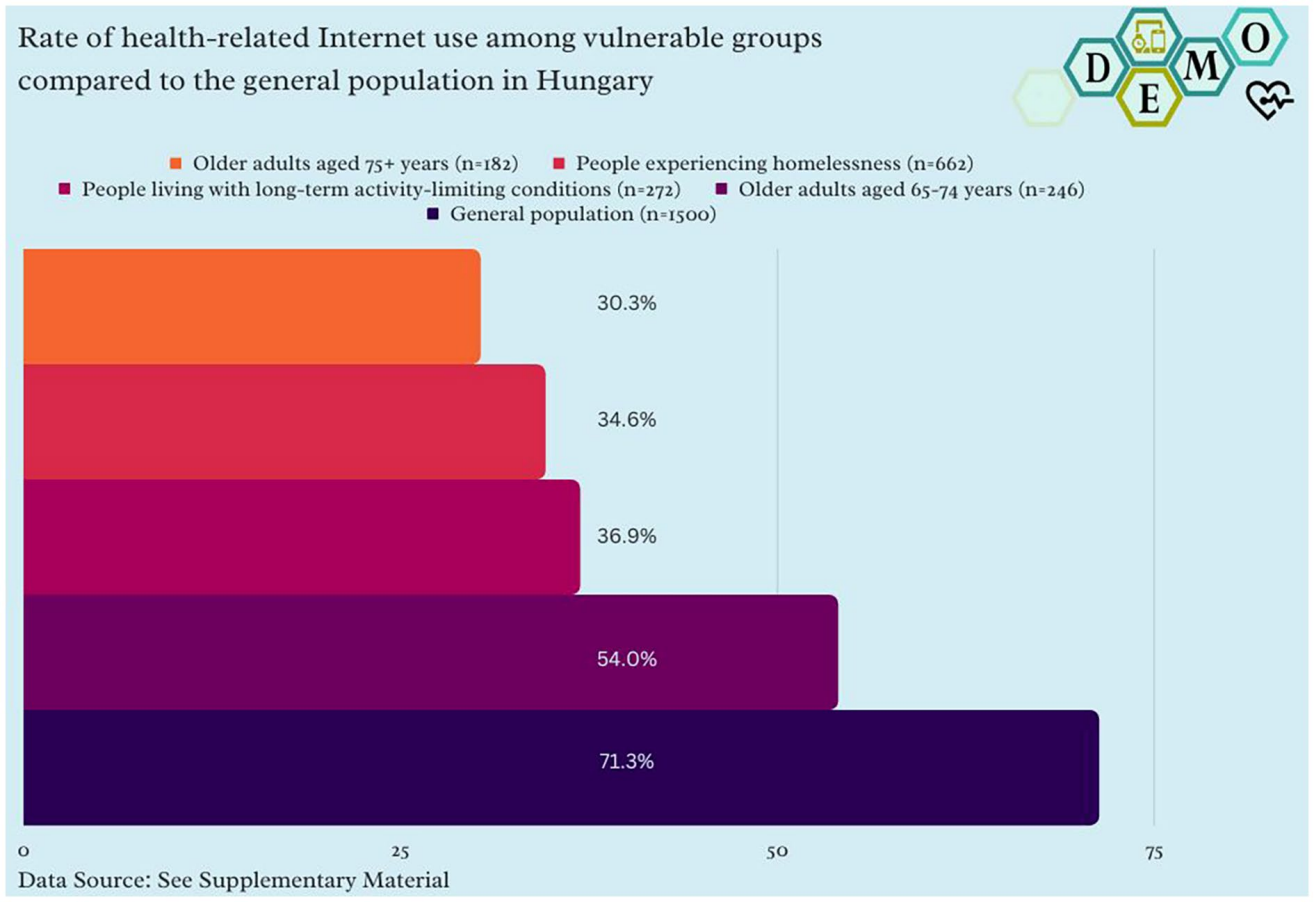
Rate of health-related Internet use among vulnerable groups compared to the general population in Hungary.

The rate of health-related Internet use among the three studied groups and the general population is summarized in Figure 1.

Methodological explainer: “Yes” responses (%) in a representative survey (n = 1,500) in Hungary in vulnerable groups and “Yes” responses (%) of people experiencing homelessness in Budapest, Hungary in a survey (n = 662) for the question: “Do you use the Internet for health-related reasons?”

### 3.3. Pillar 4: sustained engagement with the technology

Our research showed that regular Internet use of older adults for health-related purposes meant mostly accessing websites. One in two older respondents received support from family and friends in finding their way around the web. There were almost no people under 65 (0.7%), a small proportion of people aged 65–74 (2.1%), and one in 12 people aged 75+ who had never heard of any digital health solutions. Our results showed that about 70% of respondents in both age groups had used more than one digital health solution and almost the same proportion of respondents in the older age groups expressed a sustained need for more than one digital solution. As the results of our univariate and multivariate analyses showed, the most influencing sociodemographic factor was the level of education.

According to our results, although people living with long-term activity-limiting conditions showed a definite interest in digital health solutions (almost half of them would like to use various digital health technologies like smartphone telemonitoring (43.8%) or teleconsultation (40.5%)), the regular use of these technologies was only 18.7 and 4.5%, respectively.

The telemedicine pilot project supporting people experiencing homelessness was the first attempt in Hungary to test the feasibility of online visits and measure the recurrent compliance of clients. During the pilot, minimal dropout was seen, and a significant fraction (73.3%) of clients were committed to completing the full course of six telemedicine visits. Satisfaction among doctors and patients was similarly high (4.52 and 4.79, respectively, on a 5-point Likert scale). Also, a follow-up survey revealed that homeless clients were committed to utilizing telemedicine on a regular basis. The telecare setup was reinforced with institutional digital infrastructure and on-site telecare assistants recruited from social workers who assisted the patients throughout the whole process in the shelters.

## 4. Discussion: strategic considerations

Digital literacy and Internet connectivity have been called the super social determinants of health (22) as emerging new technologies represent an ever-larger part of healthcare systems in developed countries. Digital inclusion is also an EU-wide goal of the European Commission (23).

As the results of our studies demonstrated, vulnerable populations such as older adults, people living with activity-limiting conditions, and people experiencing homelessness all use digital health solutions less than the general population in Hungary. For all three groups analyzed, there are severe burdens in the use of digital health solutions, although, they all expressed their unmet needs and motivation in adopting healthtech.

Factors that may facilitate medical techquity are getting more and more important in the better utilization of public services by welfare states. In the following, we provide possible strategic considerations of a techquity-enabling framework based on our own research results. The first step is the proper scientific identification of the existing disparities. Usually, vulnerable populations are underrepresented in research activities with a medical or technological focus based on the aspects that they are either hardly reachable (e.g., people experiencing homelessness) or do not represent a desirable clientele for for-profit developments. This discrepancy needs to be addressed by a broad collaboration of healthcare and social stakeholders, and policymakers by reinforcing research activities in this field.

In the development and management of digital health services, the internalization of different aspects of diversity and inclusion is essential. This cannot be done without ensuring that technology developers, healthcare providers, and regulatory bodies represent diverse perspectives and experiences, and novel technology is designed to cover the needs of underserved individuals. In their case, person-centered care involves designing healthcare services and technology that are tailored to unique needs that might differ from the general population’s preferences. People living with long-term activity-limiting health impairments found fewer advantages in digital health solutions although our data indicated that they were interested in them. Such barriers have to be addressed by participatory design and providing technologies that people with speech, visual, hearing, or manual impairments can utilize without limitations as well.

To promote techquity, technology needs to be affordable to everybody regardless of socioeconomic status to reach universal digital health coverage. While the literature shows that as telehealth evolves, seniors are open to using it, there are underlying barriers related not only to the lack of knowledge or confidence but to costs as well (24). Among younger people, the financial situation did not affect Internet use: there was no difference among people of different socioeconomic statuses. In the case of the older adult, however, more educated individuals in a better financial situation used the Internet more. This indicated that Internet use among them was both an economic and cultural question that can be addressed by providing training opportunities, user-friendly programs, and subsidized (low-cost or free) technology through a direct financial aid (25).

Also, community initiatives, peer support, and social organizations play a critical role in promoting digital health technology in the social sector as they often dispose of a better understanding of the needs and challenges of underserved populations. Peer learning and community-level support are not only recognized by the literature, but it was also a significant result of our research (26). More seniors who received help from family and friends became regular Internet users. Also, in homeless shelters, a combination of trust and institutional support ensured the successful adoption of digital health solutions. On-site social workers as trusted intermediaries served not only as technical support but catalysts of care continuity leading to sustained use even for those who were not digitally engaged beforehand. For the closer integration of care systems, digital health solutions as umbrella services should be built up in the social sector. We recommend a public-private partnership to design a comprehensive strategy for better inclusion of vulnerable populations (older adult, people with long-term activity-limiting conditions, and people experiencing homelessness) in digital health. For people experiencing homelessness, we suggest improving digital infrastructure by setting up free Wi-Fi hotspots and phone payment schemes in city centers. Digital health literacy programs involving shelters, specialists, and government agents should also be established. Collaboration between shelter workers, educators, and digitally engaged homeless individuals can enhance their digital health learning. A similar approach can be applied to older individuals and those with activity-limiting conditions. A partnership with telecom companies can provide them with phone payment schemes and tailored tools. Additionally, digital health literacy programs involving specialists, government agents, health institutions, and pensioners’ clubs can be developed, with the option to engage digitally active older adult individuals in the learning process.

Our recommendations are in line with the five A’s of access for techquity (27). These are covering the following: availability (existence of services vs. clients’ needs), accessibility (skills, literacy, and support), accommodation (platform requirements and the ability to navigate), affordability (costs and the ability to pay), acceptability (clients’ attitude and comfort of use). Systematic implementation of these strategies might work toward achieving techquity, a goal worthy of pursuit by policymakers interested in combating health inequalities. However, providers and institutions covering vulnerable groups should not be left alone in fighting inequalities and need significant structural support. A healthcare ecosystem with a strong digital component could more successfully reduce the disadvantageous effects of a permacrisis. In many areas of society, reserve capacities such as buffers, redundancies, and insurance can improve a system’s resilient capacity to face disruptions (28). An equitable digital health ecosystem would represent this kind of strong reserve in case of any systemic breakdowns such as the ones experienced during the COVID-19 pandemic waves for the most vulnerable individuals.

As policy interventions, both national programs and targeted, vulnerability-specific approaches are needed. As an example, the National Digitalization Strategy (NDS) 2021–2030 is a governmental effort to speed up digital development in Hungary (29). The pillars of the strategy are digital infrastructure, digital skills, digital economy, and digital state. Among other actions, large-scale programs are set up highlighting interventions for social inclusion and digital health (improving the competence of both citizens and healthcare workers). As part of the program, digital devices were also allocated to people in need of it. These might have positive effects on multiple vulnerable groups, however, do not replace specific solutions tailored to marginalized groups (e.g., digital harm reduction tools for people experiencing homelessness). Local communities can also translate central initiatives to local needs better.

## Data availability statement

The raw data supporting the conclusions of this article will be made available by the authors, without undue reservation.

## Ethics statement

All our studies were approved by the Research Ethics Committee of the Hungarian Medical Research Council. The ethical license number is IV-10927-1/EKU. The patients/participants provided their written informed consent to participate in this study.

## Author contributions

ZG and SB were responsible for developing the theoretical framework, interpreting the data, and writing the manuscript. NR, JB, and BD provided and interpreted the data. All authors discussed the results and contributed to the final manuscript.

## Funding

The research project was supported by the National Research, Development and Innovation Office (OTKA-FK 134372).

## Conflict of interest

SB was employed by FitPuli Kft.

The remaining authors declare that the research was conducted in the absence of any commercial or financial relationships that could be construed as a potential conflict of interest.

## Publisher’s note

All claims expressed in this article are solely those of the authors and do not necessarily represent those of their affiliated organizations, or those of the publisher, the editors and the reviewers. Any product that may be evaluated in this article, or claim that may be made by its manufacturer, is not guaranteed or endorsed by the publisher.
