# Supplementary material for: State of vulnerable populations in the techquity framework in Hungary
Source: Front Public Health. 2023 Jul 6;11:1215325. doi: 10.3389/fpubh.2023.1215325 (PMC10357289; doi:10.3389/fpubh.2023.1215325)
Supplement: Supplementary file 1 [file Data_Sheet_1.PDF]

Supplementary Material

State of Vulnerable Populations in the Techquity Framework in Hungary

Zsuzsa Győrffy*, **Bence Döbrössy, Nóra Radó, Sándor Békási**

*** Correspondence:** Zsuzsa Győrffy: gyorffy.zsuzsanna@med.semmelweis-univ.hu

Supplementary Data

Table 1 – Socio-demographical distribution of older adults as seen in the nationwide, representative "E-patients in Hungary" population survey of 1,500 people. Data collection took place between October 5-13, 2021 lead by the Digital Health Work Group of the Institute of Behavioral Sciences, Semmelweis University. The research was implemented under the OTKA -FK 134372 basic program of Hungary.

Girasek, E., Boros, J., Döbrössy, B., Susánszky, A., & Győrffy, Z. (2022). E-páciensek Magyarországon: Digitális egészséggel kapcsolatos ismeretek, szokások egy országos reprezentatív felmérés tükrében, *Orvosi Hetilap*, *163*(29), 1159-1165. doi: https://doi.org/10.1556/650.2022.32512

**Demographics**		**18 - 64 years old**	**65-74 years old**	**75+ years old**
**Gender**				
Male	n	651	100	51
	%	50,3%	40,7%	28,0%
Female	n	643	146	131
	%	49,7%	59,3%	72,0%
Total	n	1294	246	182
**Level of education**				
Elementary school or lower	n	231	136	107
	%	17,8%	55,5%	58,8%
Vocational school / skilled worker	n	348	27	12
	%	26,9%	11,0%	6,6%
High school	n	456	51	44
	%	35,2%	20,8%	24,2%

Higher education	n	260	31	19
	%	20,1%	12,7%	10,4%
Total	n	1295	245	182
**Type of residence**				
Budapest	n	223	41	47
	%	17,2%	16,7%	25,8%
County capital, large city	n	240	41	29
	%	18,5%	16,7%	15,9%
Other city	n	459	85	59
	%	35,5%	34,7%	32,4%
Village	n	372	78	47
	%	28,7%	31,8%	25,8%
Total	n	1294	245	182

Table 2 – Socio-demographical distribution of people living with long-term activity limiting conditions as seen in the nationwide, representative "E-patients in Hungary" population survey of 1,500 people. Data collection took place between October 5-13, 2021 lead by the Digital Health Work Group of the Institute of Behavioral Sciences, Semmelweis University. The research was implemented under the OTKA -FK 134372 basic program of Hungary.

Boros J, Girasek E, Döbrössy B, Gyorffy Z. Use of digital healthcare among people living with disabilities. Hungarian Journal of Disability Studies & Special Education 2022;2022/2 (Special Issue). doi:DOI 10.3128

**Key Demographics**	**Representative sample**		**People living with long-term activity-limiting conditions**
Male	n	586	99
	%	48.1%	36.7%
Female	n	633	173
	%	51.9%	63.6%
Total	n	1219	272
Elementary school or lower	n	274	136
	%	22.4%	50.1%
	n	292	42

Vocational school / skilled worker	%	23.9%	16.7%
High school	n	415	65
	%	34%	23.9%
Higher education	n	239	28
	%	19.6%	10.3%
Total	n	1220	271
Budapest	n	230	40
	%	18.9%	14.6%
County capital, large city	n	223	45
	%	18.3%	16.4%
Other city	n	431	93
	%	35.3%	34%
Village	n	336	95
	%	27.5%	34.7%
Total	n	1220	273
18-29-year-olds	n	249	22
	%	20.4%	8%
30-39-year-olds	n	275	20
	%	22.6%	7.3%
40-49-year-olds	n	214	24
	%	17.6%	8.8%
50-59-year-olds	n	215	47
	%	17.6%	17.2%
Above 60 years	n	266	159
	%	21%	58.7%
Total	n	1219	272

Table 3 – Socio-demographical distribution of people experiencing homelessness as measured in Győrffy Z, Békási S, Döbrössy B, Bognár VK, Radó N, Morva E, et al. (2022) Exploratory attitude survey of homeless persons regarding telecare services in shelters providing mid- and long-term accommodation: The importance of trust. PLoS ONE 17(1): e0261145. https://doi.org/10.1371/journal.pone.0261145

For comparative analysis, in the case of the reference group, correctional weighting procedure was also executed in order to match it with the homeless group: gender and educational level weights were calculated and used in the following comparative analysis.

Key Demographics		Homeless persons	Reference population	Reference population (weighted)
Male	n	89	61	103
	%	90.8%	55.5%	92%
Female	n	9	49	9
	%	9.2%	44.5%	8%
Total	n	98	110	112
Less than 8 years of elementary school	n	3	3	4
	%	3.1%	2.7%	3.6%
Elementary school	n	25	6	19
	%	25.8%	5.5%	17%
Vocational school	n	48	25	69
	%	49.5%	22.7%	61.6%
High school	n	15	29	14
	%	15.5%	26.4%	12.5%
Higher education	n	6	47	6
	%	6.2%	42.7%	5.4%
Total	n	91	100	112
18-29-year-olds	n	249	12	22
	%	20.4%	10.9%	8%
30-39-year-olds	n	275	17	20
	%	22.6%	15.4%	7.3%
40-49-year-olds	n	214	19	24
	%	17.6%	17.2%	8.8%
50-59-year-olds	n	215	28	47
	%	17.6%	25.4	17.2%
Above 60 years	n	266	34	159
	%	21%	30.9%	58.7%
Total	n	98	110	112

Table 4 – Socio-demographical distribution of people experiencing homelessness as measured in Radó N, Girasek E, Békási S, Győrffy Z. *Digital Technology Access and Health-Related Internet Use Among People Experiencing Homelessness in Hungary: Quantitative Survey*. J Med Internet Res 2022;24(10):e38729

The representative sample indicates the sample in the nationwide, representative "E-patients in Hungary" population survey of 1,500 people mentioned above.

**Key Demographics**	**Homeless persons**		**Representative sample**
Male	n	459	586
	%	71.2%	48.1%
Female	n	203	633
	%	28.8%	51.9%
Total	n	662	1219
Elementary school or lower	n	252	274
	%	38.1%	22.4%
Vocational school	n	232	292
	%	35%	23.9%
High school	n	135	415
	%	20.4%	34%
Higher education	n	30	239
	%	4.5%	19.6%
Total	n	662	1220
18-44-year-olds	n	165	738
	%	25.9%	60.6%
50-59-year-olds	n	233	215
	%	35.3%	17.6%
Above 60 years	n	247	266
	%	38.8%	21%
Total	n	650	1220
